# The Walker 256 Breast Cancer Cell- Induced Bone Pain Model in Rats

**DOI:** 10.3389/fphar.2016.00286

**Published:** 2016-08-31

**Authors:** Priyank A. Shenoy, Andy Kuo, Irina Vetter, Maree T. Smith

**Affiliations:** ^1^School of Biomedical Sciences, The University of QueenslandBrisbane, QLD, Australia; ^2^Centre for Integrated Preclinical Drug Development, The University of QueenslandBrisbane, QLD, Australia; ^3^Institute for Molecular Bioscience, The University of QueenslandBrisbane, QLD, Australia; ^4^School of Pharmacy, The University of QueenslandBrisbane, QLD, Australia

**Keywords:** breast cancer, metastasis, bone pain, Walker 256 cell, rat model

## Abstract

The majority of patients with terminal breast cancer show signs of bone metastasis, the most common cause of pain in cancer. Clinically available drug treatment options for the relief of cancer-associated bone pain are limited due to either inadequate pain relief and/or dose-limiting side-effects. One of the major hurdles in understanding the mechanism by which breast cancer causes pain after metastasis to the bones is the lack of suitable preclinical models. Until the late twentieth century, all animal models of cancer induced bone pain involved systemic injection of cancer cells into animals, which caused severe deterioration of animal health due to widespread metastasis. In this mini-review we have discussed details of a recently developed and highly efficient preclinical model of breast cancer induced bone pain: Walker 256 cancer cell- induced bone pain in rats. The model involves direct localized injection of cancer cells into a single tibia in rats, which avoids widespread metastasis of cancer cells and hence animals maintain good health throughout the experimental period. This model closely mimics the human pathophysiology of breast cancer induced bone pain and has great potential to aid in the process of drug discovery for treating this intractable pain condition.

## Introduction

The most common cause of pain in cancer arises from bone metastasis, and around 73% of patients with terminal breast cancer exhibit indications of bone metastases (Coleman, 2006; Currie et al., 2013; Bu et al., 2014). Of these, 75% suffer severe bone pain and pathological fractures (Ibrahim et al., 2013). This is in contrast to primary breast tumors in the tissue of origin that cause very little or no pain at all (Lozano-Ondoua et al., 2013). Clinically, nonsteroidal anti-inflammatory drugs are the mainstay of treatment, often in combination with strong opioid analgesics, radiotherapy in the initial stages of metastasis, and adjuvant agents that inhibit osteoclast activity such as bisphosphonates and denosumab (Mantyh et al., 2002; Colvin and Fallon, 2008; Fallon et al., 2016; Fernandes et al., 2016). The principal challenge in understanding the pathophysiological mechanisms of cancer-induced bone pain (CIBP) is the development of an animal model which has characteristics in common with that of human CIBP (Slosky et al., 2015). It is only recently that preclinical studies have begun to determine how metastatic cancers may interact with the bone microenvironment resulting in pain (Lozano-Ondoua et al., 2013). Until the late twentieth century, all animal models of CIBP relied on systemic injection of carcinoma cells, which resulted in poor animal health because of metastases in vital organs such as the liver, lungs, brain, and multiple sites in bone (Urch, 2004; Simmons et al., 2015). Subsequently, the more efficient method of local infusion of cancer cells into a single bone was developed, thereby avoiding systemic spread of cancer cells and the maintenance of good general animal health (Schwei et al., 1999). Although, multiple breast cancer cell lines have been used to induce bone tumors in rats and mice, the focus of this mini-review is confined to research in which Walker 256 rat breast cancer cells have been used to induce bone pain in rats.

## Rat as the species of choice

Rats and mice are the most commonly used animal species for pain research (Walker et al., 1999), with rats being superior to mice in many practical respects (Wilson and Mogil, 2001; Mogil, 2009). The advantage of mouse pain models is the availability of transgenic mice for dissecting pathophysiological mechanisms (Mogil and Grisel, 1998) and mouse models of breast cancer might recapitulate key aspects of human breast cancer including poor immunogenicity and high metastatic potential (Hahn et al., 2006). However, the main disadvantage of mice is their small size, making direct injection of tumor cells into the bone technically challenging (Pacharinsak and Beitz, 2008). By contrast, rat models are considered very suitable for efficacy assessment of therapeutic interventions for the treatment of breast CIBP (Medhurst et al., 2002). The model using Walker 256 cells can be induced in both sexes of rats (Liu et al., 2010, 2011) and different rat strains are compatible with these cells (Earle, 1935; Jensen and Muntzing, 1970). Stage of the estrous cycle in female rats does not alter the development of CIBP (Zhu G. Q. et al., 2014).

## Suitability of walker 256 cells

The Walker tumor was first discovered in the breast of a pregnant albino rat (*Rattus norvegicus*) in 1928 by Dr. George Walker in Baltimore and it is regarded as a carcinosarcoma (McEuen and Thomson, 1933; Simpkins et al., 1991). It is one of the most widely used transplantable tumors in experimental research (Justice, 1985; Brigatte et al., 2007; Fan et al., 2016; Gambeta et al., 2016; Gao et al., 2016; Sroka et al., 2016; Wu M. et al., 2016). Indeed, these cells are one of the most preferred cell lines because of the ease with which they can be standardized, maintained and propagated *in vitro*, as well as their extensive use *in vivo* since 1937 (Michaelson and Orcutt, 1957; Brigatte et al., 2016; Galuppo et al., 2016; Pigatto et al., 2016; Trashkov et al., 2016; Yalovenko et al., 2016).

Walker 256 cells cause significant bone resorption and increase skeletal fragility at the site of implantation in rats (Kurth et al., 2000), consistent with the phenotype observed in breast cancer patients with bone metastasis (Shih et al., 2004). In addition to being a reproducible method for inducing skeletal metastasis (Blouin et al., 2005; Mao-Ying et al., 2006; Badraoui et al., 2009), this model mimics key features of human breast CIBP, including pharmacological profile (Mao-Ying et al., 2006, 2012; Cao et al., 2010). Walker 256 cells can be used in a variety of rat strains (Hang et al., 2015; Lu et al., 2015) because these cells produce uniformly rapid growth, show very little regression, and are readily adaptable (Lewis et al., 2013; Oliveira and Gomes-Marcondes, 2016).

Growth of Walker 256 cells in the form of tumor is practically independent of the age and weight of the animals at the time of their inoculation (Walpole, 1951). Another advantage is that after unilateral intra-tibial injection (ITI), tumor cells do not metastasize to the contralateral tibia during the experimental period and they only cause structural degradation of bones in the ipsilateral limb but not the contralateral limb (Kurth et al., 2001, 2002). They also generally do not metastasize to highly perfused organs such as the lungs (Brigatte et al., 2007), in contrast to other cell lines such as the 13762 rat mammary carcinoma cell line or the c-SST2 rat mammary carcinoma cell line, which spontaneously metastasize (Blouin et al., 2005).

Although, many scientists tend to presume that tumor cell lines behave indefinitely in a uniform manner (Lewis et al., 2013), changes may be induced by factors such as extended *in vitro* growth time, high passage number and cross contamination with other cell lines (Sacchi et al., 1984; Chang-Liu and Woloschak, 1997; Buehring et al., 2004; Liscovitch and Ravid, 2007). Immortalized cancer cell lines may also evolve *in vivo* over time in the animal models in which cancer is induced (Poste et al., 1982b). Various heterogeneous subpopulations of tumor cells within a tumor mass possess diverse metastatic potential and different propensities for metastasis to various organs (Fidler, 1978; Poste et al., 1982a). Similarly, immortalized Walker 256 cancer cell lines from different cell banks may possess diverse characteristics and behavior *in vivo* despite the fact that these cell lines are from rat origin and are without contamination (Lewis et al., 2013). In general, cell lines may be authenticated by short tandem repeat (STR) profiling of the microsatellite regions of DNA (Nims et al., 2010). However, as there is no reference DNA profile of the Walker 256 cell line (Lewis et al., 2013), researchers typically procure cells of a defined passage number from reputable cell banks. To minimize within- and between- laboratory variability in the use of these cells *in vivo*, it is important that cultured cells are banked and frozen at early passages, and that culture conditions including growth media, temperature, humidity and exposure to drugs are standardized (Marx, 2014).

## General methodology

Although there are minor between-laboratory variations, the general method for induction of breast CIBP in rats has several aspects in common. The procedure generally involves making an incision to the skin and muscle around the knee joint of the anesthetized rat and injecting cancer cells into the tibial bone, followed by sealing of the drilled hole with bone wax, suturing of the wound and close monitoring of animals during post-surgical recovery (Mao-Ying et al., 2006). Cells can also be injected in the femur (Gui et al., 2013, 2015). Small differences in the number of injected Walker 256 cancer cells due to experimental errors typically have a minimal effect on the study outcome (Kurth et al., 2001). The physical process for injection of Walker 256 cells into the medullary canal of the bone does not impact the study outcome adversely as emphasized by the normal fibroblastic healing response around the drilled hole of injected bone (Kurth et al., 2002; Mao-Ying et al., 2006). Although outflow of cells during the injection process can be a common occurrence associated with the model, the syringe can be left in place inside the medullary canal of the bone for an additional 1 or 2 min to avoid leakage of cells along the injection track (Mao-Ying et al., 2006; Yu et al., 2009; Miao et al., 2010; Dong et al., 2011; Hu S. et al., 2012).

## Time frame for development of pain behaviors and analgesic efficacy testing

One of the most important and critical factors in the study of pain behavior and extent of bone destruction in this model is the timing of observations post-surgery (Qiu et al., 2012). Large tumors can develop in just a few days (Justice, 1985). However, the time period for development of pain behaviors may vary between studies based upon factors such as cell invasiveness and sex of the experimental animals (Wang et al., 2011). Pain behavior due to the surgical process may be evoked in the ipsilateral (injected) hind paws if the animals are tested immediately after the inoculation surgery (Lan et al., 2010; Dong et al., 2011). Hence a recovery period of 2–3 days post-surgery must be provided for the animals (Wang et al., 2011). For the purposes of studying different mechanisms of breast CIBP and for efficacy profiling of molecules with potential to be developed as novel analgesic agents, it is best to avoid extending the model beyond 20–25 days post-surgery (Mao-Ying et al., 2006; Yu et al., 2009; Cao et al., 2010; Tong et al., 2010; Hang et al., 2014) due to overall poor animal health and ethical concerns (Kurth et al., 2001). In particular, prolonged observation times may be associated with more complex pathophysiology arising from systemic metastasis due to severe osteolysis (Qiu et al., 2012). Hence, the period between days 6 and 18 post-ITI is typically chosen for investigation of breast CIBP mechanisms and the efficacy testing of novel compounds with potential as analgesic agents (Wang et al., 2011; Hu et al., 2012a; Wang L. N. et al., 2012b).

## Nature of pain manifestation

In Walker 256 cell-CIBP, up-regulated expression and release of pro-inflammatory mediators including prostaglandin E2 (PGE2), nerve growth factor (NGF), and proinflammatory cytokines including interleukin (IL)-1β, IL-6 and tumor necrosis factor-α (TNF-α) in the spinal cord and dorsal root ganglia contributes to the pathogenesis of bone pain in rats (Cao et al., 2010; Lan et al., 2010; Liu et al., 2010; Dong et al., 2011; Mao-Ying et al., 2012; Yao et al., 2016; Zhu et al., 2016). Hence, neuroinflammation is an important pathogenic characteristic of this model (Hu S. et al., 2012; Song et al., 2015).

Similar to the clinical situation, Walker 256 cell-CIBP manifests as spontaneous pain, hyperalgesia, allodynia as well as ambulatory pain, the severity of which largely depends upon the number of inoculated cells, but can also be affected by other experimental factors including cell origin as well as strain or sex of the animals used (Mao-Ying et al., 2006; Liu et al., 2010). Similarly, hind paw hypersensitivity induced by ITI with Walker 256 cells may be either unilateral (Liu et al., 2010; Tong et al., 2010; Dong et al., 2011; Wang J. et al., 2012; Wang L. N. et al., 2012b) or bilateral (Mao-Ying et al., 2006, 2012; Zhao et al., 2013; Li et al., 2014). Peripheral mechanisms including circulating factors and transmedian sprouting, or central mechanisms such as signaling via commissural interneurons in the spinal cord and brain stem may underpin unilateral injury-induced contralateral mirror effects (Koltzenburg et al., 1999). This mirror image effect may also be correlated with spinal glia cell activation, proinflammatory cytokine production, and morphological changes within the local nerve, suggesting the involvement of glia (Chacur et al., 2001). The mirror image pain behavior induced in the contralateral hind paw in this model may be observed when the tumors are in the advanced stage (Miao et al., 2010; Zhao et al., 2013; Li et al., 2014) Typically though, contralateral pain behaviors are of reduced intensity compared with the ipsilateral hind paw (Miao et al., 2010).

Thermal and mechanical pain behaviors are underpinned by different mechanisms (Paqueron et al., 2003; Wang J. et al., 2012). Cutaneous nociceptors are particularly sensitized by thermal stimuli and nociceptors present in deep somatic tissues such as joints and muscle exhibit pronounced sensitization to mechanical stimuli (Schaible, 2007). Although, thermal hyperalgesia has been reported in this model (Liu et al., 2011; Duan et al., 2012; Wang J. et al., 2012), there are several studies in which hindpaw hypersensitivity to an applied noxious heat stimulus is not observed in rats following a unilateral ITI of Walker 256 cells (Mao-Ying et al., 2006, 2012; Yao et al., 2008; Miao et al., 2010; Wang et al., 2011). Again, these differences may be attributed to various factors including between-vendor differences in animals and cancer cell-related factors. For this reason, thermal hyperalgesia is not typically used as a pain behavioral endpoint in this model (Yu et al., 2009; Cao et al., 2010; Tong et al., 2010; Zhao et al., 2010; Dong et al., 2011). A between-study comparison of Walker 256 cell- CIBP rat model is presented in Table 1.

**Table 1:** **Comparative summary of previous work by others using the Walker 256 cell-CIBP model in rats**.

**Number of cells injected**	**Rat Sex, Strain- (Number of studies)**	**Time frame of hind paw hypersensitivity post- ITI (Days)**	**Nature of pain behavioral responses in the hind paw**				**References**
			**MA**	**MH**	**TH**	**S/MEP**	
4 × 10^3^	F, W- (1)	14–19	+	NA	NA	+	Cao et al., 2010
5 × 10^3^	F, SD- (1)	6–14	+	NA	NA	NA	Zhao et al., 2013
1 × 10^4^	F, W- (1)	9–21	+	NA	NA	+	Ke et al., 2013
1 × 10^4^	F, SD- (1)	7–18	+	NA	+	+	Yao et al., 2016
3 × 10^4^	F, SD- (1)	Tested day 10	+	NA	NA	NA	Liu et al., 2012
4 × 10^4^	F, W- (3)	3–16	+	NA	NA	NA	Dong et al., 2011; Bu et al., 2014; Xia et al., 2014; Ye et al., 2014; Guan et al., 2015
	F, SD- (2)						
5 × 10^4^	F, SD- (2)	7–18	+	NA	+	NA	Qiu et al., 2014; Wang et al., 2016
5 × 10^4^	F, SD- (1)	5–14	NA	+	+	NA	Qiu et al., 2012
1 × 10^5^	F, W- (2)	12–24	+	NA	+	+	Bao et al., 2014b, 2015c
1 × 10^5^	M&F, SD- (4)	5–28	+	NA	+	NA	Liu et al., 2011; Jiang et al., 2014; Bao et al., 2015a; Fan et al., 2015; Jiang et al., 2015; Ren et al., 2015; Jiang et al., 2016
	M&F, W- (3)						
1 × 10^5^	F, SD- (8)	6–21	+	NA	NA	+	Lan et al., 2010; Liu et al., 2010; Chen et al., 2012; Hang et al., 2012; Wang L. N. et al., 2012a,b; Jin et al., 2014; Bian et al., 2016
1 × 10^5^	F, SD- (8)	5–21	+	NA	NA	NA	Wang et al., 2011; Hu et al., 2012a,b; Hang et al., 2013b,c; Hu et al., 2013; Hang et al., 2015, 2016; Zhu et al., 2015
	F, W- (1)						
1 × 10^5^	F, SD- (1)	6–15	NA	+	NA	+	Hang et al., 2014
1 × 10^5^	F, SD- (1)	6–15	NA	+	NA	NA	Hang et al., 2013a
1 × 10^5^	F, W- (1)	Tested day 14	NA	NA	NA	+	Bao et al., 2015b
2 × 10^5^	F, SD- (2)	3–21	+	NA	NA	NA	Huang et al., 2014; Jin et al., 2015; Pan R. et al., 2015; Wu J. X. et al., 2016
	F, W- (1)						
2 × 10^5^	F, W- (1)	7–21	NA	+	−	+	Miao et al., 2010
2 × 10^5^	F, SD- (1)	7–25	NA	+	−	NA	Li et al., 2014
2 × 10^5^	F, W- (1)	7–21	NA	+	+	+	Wu et al., 2012
3.5 × 10^5^	F, SD- (2)	5–21	+	NA	+	NA	Wang J. et al., 2012; Wang et al., 2015
4 × 10^5^	F, SD- (1)	5–21	+	NA	−	+	Yin et al., 2010
4 × 10^5^	F, SD- (1)	4–32	+	NA	−	NA	Huang et al., 2012; Mao-Ying et al., 2012
	F, W- (1)						
4 × 10^5^	F, W- (2)	7–21	+	NA	+	+	Duan et al., 2012; Yang et al., 2015; Zhou et al., 2015
	F, SD- (1)						
4 × 10^5^	SD- (1)	3–21	+	NA	NA	+	Cheng et al., 2014
4 × 10^5^	M&F, W- (10)	3–21	+	NA	NA	NA	Yu et al., 2009; Tong et al., 2010; Hu S. et al., 2012; Wang X. W. et al., 2012a,b; Li et al., 2013; Zhang et al., 2013; Gong et al., 2014; Zhu B. et al., 2014; Hu S. et al., 2015; Li et al., 2016; Song et al., 2016
	F, SD- (2)						
4 × 10^5^	F, W- (1)	6–20	NA	NA	+	NA	Xu et al., 2013
5 × 10^5^	F, SD- (1)	7–21	+	NA	+	+	Liu et al., 2013
5 × 10^5^	F, SD- (5)	5–21	+	NA	+	NA	Bao et al., 2014a; Liu et al., 2014; Shen et al., 2014; Hu X. M. et al., 2015; Zhang et al., 2015; Zhu et al., 2016
	F, W- (1)						
5 × 10^5^	F, SD- (1)	7–10	+	NA	NA	+	Lu et al., 2015
5 × 10^5^	F, SD- (3)	9–21	+	NA	NA	NA	Chen et al., 2013, 2015; Song et al., 2015
5 × 10^5^	F, SD- (1)	7–10	NA	+	NA	+	Lu et al., 2016
5 × 10^5^	M, SD- (1)	5–14	NA	+	NA	NA	Xu et al., 2015
1 × 108	F, SD- (1)	7–25	+	NA	NA	NA	Zhao et al., 2010

*+, observed; −, not observed; F, female; M, male; MA, mechanical allodynia; MH, mechanical hyperalgesia; NA, not assessed; SD, Sprague Dawley; S/MEP, spontaneous or movement- evoked pain; TH, thermal hyperalgesia; W, Wistar*.

## Regression of tumor and resolution of pain

Similar to the well-known human scenario of breast cancer regression (Lewison, 1976; Hutter, 1982; Burnside et al., 2006; Barry, 2009; Onuigbo, 2012), Walker 256 breast cancer cells may also potentially transform into a regressive variant *in vivo* (Guimarães et al., 2010) resulting in complete regression if the study is prolonged (Jensen and Muntzing, 1970; Cavalcanti et al., 2003; Schanoski et al., 2004). The mechanisms underlying spontaneous regression are not entirely clear but may involve development of an adaptive immune response (Pardoll and Topalian, 1998; Rees and Mian, 1999), differential propagation of tumor sub clones in their microenvironment (Khong and Restifo, 2002) and consequent elimination by immune cells, antibodies, cytokines, and chemokines (Dunn et al., 2002, 2006; Bui and Schreiber, 2007; Jaganjac et al., 2008). Physical activity of the animals, exercise (Hoffman et al., 1962; Deminice et al., 2016b), dietary factors (Bekesi and Winzler, 1970; Kwong et al., 1984; Luty et al., 2016), or hormonal levels (Khegai, 2013; Khegay and Ivanova, 2015) may influence the regression of these cells or inhibit the activities driven by these cells (Campos-Ferraz et al., 2016; Cruz et al., 2016; Deminice et al., 2016a; Fracaro et al., 2016; Toneto et al., 2016). In most studies, tumor regression is generally overlooked as the tumor-bearing rats are sacrificed before regression is evident (Guimarães et al., 2010). Thus, the verification of tibial tumor burden post-mortem is very important. However, the beginning of pain behavior resolution at 20–25 days post-surgery is typically not due to tumor regression, but may involve neuro-immune mechanisms (Zhao et al., 2010; Xu et al., 2013; Huang et al., 2014). In previous work by others using different cancer cell lines, up regulation of the endogenous opioid system is implicated in spontaneous pain behavior resolution (Muralidharan et al., 2013). Similarly, endogenous opioid system could also have a role in Walker 256 cell-CIBP model (Li et al., 2016). In addition, lipoxins and endogenous lipoxygenase-derived eicosanoids, which represent a unique class of lipid mediators, have a broad spectrum of anti-inflammatory and antinociceptive activities. These are known to suppress the expression of spinal pro-inflammatory cytokines and might also contribute to spontaneous resolution of Walker 256 cell-CIBP in rats (Hu S. et al., 2012). Inflammation, which is an important component of cancer pain (Falk and Dickenson, 2014) mostly involves active endogenous processes targeted at protecting the host, and is generally self-limiting and self-resolving (Chiang et al., 2005; Serhan and Savill, 2005; Schwab and Serhan, 2006).

## Targets for novel analgesic drug discovery

The pathobiology of Walker 256 cell-CIBP in rats is complex involving inflammatory, neuropathic and tumorigenic components (Cao et al., 2010). Following injection, these cells cause osteolysis and bone resorption (Kurth et al., 2000, 2001; Yu et al., 2009) and increase oxidative stress and impair the antioxidant system in the bone microenvironment (Badraoui et al., 2009). They cause enhanced synthesis of IL-1β and TNF-α at the mRNA or protein level along with nuclear factor kappa-light-chain-enhancer of activated B cells (NF-κB), which indicates that increased neuroimmune responses is one of the important factors responsible for pain in this model (Cao et al., 2010; Song et al., 2016). Injection of these cells in the bones sequentially activates the extracellular signal-regulated protein kinase (ERK)/mitogen-activated protein kinase (MAPK) pathway in various cell types in the spinal cord of rats (Wang et al., 2011; Wang X. W. et al., 2012b; Bian et al., 2016). Sodium channels expressed by sensory nerve fibers including voltage gated sodium ion channels (Na_v_)1.7, Na_v_1.8, and Na_v_1.9 (Miao et al., 2010; Qiu et al., 2012; Pan J. et al., 2015) as well as potassium ion channels, high-voltage-activated calcium channels, hyperpolarization-activated cation channels, transient receptor potential cation channel subfamily V member 1 (TRPV1) (Duan et al., 2012; Xu et al., 2013; Xia et al., 2014), and acid-sensing ion channel 3 (Qiu et al., 2014) may also be important determinants of enhanced neuronal excitability in this breast CIBP model in rats.

Pain behavior and its relief in Walker 256 cell-CIBP in rats is mediated by the endogenous effectors of several targets interacting with their cognate receptors as summarized in Table 2. These include important targets like opioid receptors, toll like receptors, chemokine receptors, and purinergic receptors.

**Table 2:** **Role of endogenous effectors interacting with their cognate targets that mediate pain and analgesia in the Walker 256 cell- CIBP model in rats**.

**Receptor**	**Ligand**	**Downstream molecule/effector**	***In vivo* pharmacological modulator used**	**References**
Toll like receptor 4 (TLR4)	Lipopolysaccharide (Saitoh et al., 2004)	TNF- α, IL-1β; IL-6; p38MAPK	Inducible Lentivirus-Mediated small interfering RNA (siRNA) against TLR4; p38MAPK inhibitor- SB203580; TLR4 blocker- lipopolysaccharide Rhodobacter sphaeroides (LPSRS)	Lan et al., 2010; Liu et al., 2010; Mao-Ying et al., 2012; Li et al., 2013; Liu et al., 2013; Pan R. et al., 2015
Lysophosphatidic acid 1 (LPA1) receptor	Lysophosphatidic acid	Phospholipase C, MAPK, protein kinase B (Akt) (Yung et al., 2014), Ras homolog gene family (Rho), Rho- associated protein kinase (ROCK)	LPA1 receptor blocker- VPC32183; Rho inhibitor- BoTXC3; ROCK inhibitor- Y27632	Zhao et al., 2010; Wu J. X. et al., 2016
Erythropoietin-producing human hepatocellular carcinoma receptor B1 (EphB1)	EphrinB1, EphrinB2	IL-1, IL-6 and TNF- α; Matrix metalloproteinase (MMP)-2/9	EphB1 receptor blocker- EphB1-Fc; EphB1 receptor blocker- EphB2-Fc	Dong et al., 2011; Liu et al., 2011
Epidermal growth factor-like receptor ErbB2	Neuregulin 1 (NRG1)	Akt-1, p38MAPK	ErbB2 inhibitor	Jiang et al., 2014
CX3C chemokine receptor 1 (CX3CR1)	Fractalkine	p38MAPK	Anti-CX3CR1 antibody	Yin et al., 2010; Hu et al., 2012a; Cheng et al., 2014
CC chemokine receptor-2 (CCR2)	Chemokine monocyte chemoattractant protein-1 (MCP-1)	phosphatidylinositol 3-kinase (PI3K), Akt	Anti-MCP-1 antibody; PI3K inhibitor LY294002; exogenous recombinant MCP-1; CCR2 antagonist RS102895	Hu et al., 2012b, 2013; Jin et al., 2015; Ren et al., 2015
Chemokine (C-X-C motif) receptor CXCR3	CXCL9, CXCL10, CXCL11	Phosphoinositide -3 kinase (PI3K), MAPK, Akt, ERK 1/2 (Smit et al., 2003)	Recombinant CXCL10 protein, anti-CXCL10 antibody, CXCR3 antagonist	Bu et al., 2014; Guan et al., 2015
CXC motif receptor 4 (CXCR4)	CXCL12	TNF-α, NF-κB, IL-6 and MAPKs	Anti-CXCL12 neutralizing Antibody, CXCR4 inhibitor-AMD3100, c-Jun N-terminal kinases (JNK) inhibitor SP600125, MAPK inhibitor U0126, p38 inhibitor SB503580	Shen et al., 2014; Hu X. M. et al., 2015
Purinergic P2Y1 receptor (P2Y1R)	Extracellular Adenosine triphosphate (ATP) (Webb et al., 1994)	ERK1/2	P2Y1R antagonist MRS2179	Chen et al., 2012
Purinergic P2X3 receptor (P2X3R)	Extracellular ATP	ERK (Seino et al., 2006)	P2X3 receptor antagonist- A-317491	Wu et al., 2012; Zhou et al., 2015
Purinergic P2X4 receptor (P2X4R)	Extracellular ATP (North, 2002)	p38MAPK	P2X4R siRNA	Jin et al., 2014
Purinergic P2X7 receptor (P2X7R)	ATP	IL-1β, IL-18, phosphorylated p38 (Arulkumaran et al., 2011)	inhibitor of P2X7R- Brilliant Blue G (BBG); RNA interference targeting the P2X7R	Huang et al., 2014; Yang et al., 2015
a3 glycine receptors	Glycine	Chloride current modulation (Avila et al., 2013)	siRNA targeting a3 GlyR, glycine receptor antagonist- strychnine	Zhang et al., 2013
Adenosine A1 receptor	Adenosine	Protein kinase C (PKC) (Hughes et al., 2015)	Adenosine A1 receptor antagonist- DPCPX	Chen et al., 2013
Protease-activated receptor 2 (PAR2)	Trypsin and trypsin-like proteinases	NF-κB	PAR2 antagonist- FSLLRY-NH2	Bao et al., 2014a, 2015b
Protease-activated receptor 4 (PAR4)	Thrombin	Vascular endothelial growth factor (VEGF), endostatin (Ma et al., 2005)	None	Bao et al., 2015c
Glucagon like peptide-1 receptor (GLP-1R)	Glucagon like peptide-1 (GLP-1)	Cyclic adenosine monophosphate (cAMP), protein kinase A (PKA)	GLP-1R agonists GLP-1(7–36)	Gong et al., 2014
Cannabinoid receptor type 2 (CB2)	2-arachidonoylglycerol (Basu et al., 2011)	IL-1β, IL-6, IL-18, TNF-α	CB2-selective antagonist- AM630; CB2-selective agonist- JWH-015	Lu et al., 2015, 2016
Prokineticin receptor 2 (PKR2)	Bv8 (prokineticin 2)	TNF- α	Bv8 neutralizing antibody	Hang et al., 2015
Corticotropin-releasing factor (CRF) receptor	Corticotropin-releasing factor (CRF)	PKA, NF-κB, ERK 1/2 (Taché and Million, 2015)	CRF receptor antagonist (α-helical-CRF)	Fan et al., 2015
μ-opioid receptor (MOR)	Endomorphin-2	Guanosine triphosphate (GTP), adenosine diphosphate (ADP) (Al-Hasani and Bruchas, 2011)	MOR antagonist- β-funaltrexamine (β-FNA)	Chen et al., 2015; Jiang et al., 2016; Yao et al., 2016
Sigma-1 Receptor	Tryptaminergic trace amines, as well as neuroactive steroids such as dehydroepiandrosterone (DHEA) and pregnenolone (Fontanilla et al., 2009)	Inositol trisphosphate (IP3)	Sigma-1 receptor antagonist -BD1047	Zhu et al., 2015
N-Methyl-D-Aspartate (NMDA) Receptor	Glutamate, glycine or D-serine (Hogan-Cann and Anderson, 2016)	PKA, MAPK (Zhao et al., 2016)	None	Wang L. N. et al., 2012a

## Limitations and potential improvement of the model

Like many other preclinical models, this model has short-comings which might hinder translation of promising preclinical data into successful clinical outcomes. Mostly, efficacy profiling in preclinical pain models is driven by a desire to reduce the intensity of pain behavioral readouts. However, a reduction in pain intensity is not always a good measure of the success of a pain treatment (Ballantyne and Sullivan, 2015). Pain is a subjective emotional experience and clinically, a powerful analgesic response can be elicited by placebo treatment (Kaptchuk and Miller, 2015; Tuttle et al., 2015). Hence, responses in experimental animals may not necessarily correlate with the responses expected from humans in the clinical setting. It is also necessary to remember that animals at different ages may process nociception differently (McKelvey et al., 2015) and hence, selection of the correct age of animals that suits the experimental goals may be critical.

Important factors that significantly affect pain research outcomes, such as the sex of the researchers interacting with the animals (Sorge et al., 2014) should not be overlooked in preclinical studies. Sex of the experimental animals or human subjects is a key source of variation in pro-nociceptive signaling (Wiesenfeld-Hallin, 2005; Sorge et al., 2015). In a recent large-scale gene regulatory study (Qu et al., 2015), the main findings were that men and women may require different strategies for treatment of pain, and so sex differences in pain research should not be ignored (Murphy et al., 2009; Vacca et al., 2014, 2016; Brings and Zylka, 2015; Cahill and Aswad, 2015; Ferrarelli, 2015).

There are many types of breast cancer in the clinical setting (Sharma et al., 2010) with the potential to cause pain, and the extent to which this model provides insights into these various subtypes is currently unclear. It is also important to have standardized protocols when using such preclinical models in order to minimize between-investigator and between-laboratory differences in implementation (Freedman and Gibson, 2015).

## Conclusion

Cancer-associated pain, especially intractable bone pain, is very debilitating (Kane et al., 2015). Although, this model involving ITI of Walker 256 cells in rats might not exactly mimic the metastatic spread of breast cancer to the axial skeleton in humans (Kurth et al., 2001, 2002), it provides great insights into the pathobiology and mechanisms of breast CIBP and is hence used very widely in experimental research (Du et al., 2015; Hang et al., 2015; Hu S. et al., 2015; Liu et al., 2015; Lu et al., 2015). Undoubtedly, it is one of the most suitable preclinical models for efficacy assessment of novel compounds from discovery programs aimed at identifying drugs with potential to alleviate breast CIBP in humans.

## Author contributions

All authors (PS, AK, IV, and MS) meet the essential authorship criteria required by the journal including (a) substantial contributions to the conception and design of this article; the acquisition, analysis, and interpretation of the work, (b) drafting the work and revising it critically for important intellectual content, (c) final approval of the version to be published, and (d) agreement to be accountable for all aspects of the work in ensuring that questions related to the accuracy or integrity of any part of the work are appropriately investigated and resolved.

### Conflict of interest statement

The authors declare that the research was conducted in the absence of any commercial or financial relationships that could be construed as a potential conflict of interest.

## Abbreviations

CIBP: cancer- induced bone pain
ITI: intra-tibial injection.
